# Elevated TyG Index Predicts Incidence of Contrast-Induced Nephropathy: A Retrospective Cohort Study in NSTE-ACS Patients Implanted With DESs

**DOI:** 10.3389/fendo.2022.817176

**Published:** 2022-02-22

**Authors:** Mingkang Li, Linqing Li, Yuhan Qin, Erfei Luo, Dong Wang, Yong Qiao, Chengchun Tang, Gaoliang Yan

**Affiliations:** ^1^School of Medicine, Southeast University, Nanjing, China; ^2^Department of Cardiology, Zhongda Hospital, Southeast University, Nanjing, China

**Keywords:** non-ST elevated acute coronary syndrome (NSTE-ACS), TyG index, insulin resistance, diabetes mellitus, contrast-induced nephropathy (CIN)

## Abstract

**Background:**

Triglyceride-glucose (TyG) index is a reliable and specific biomarker for insulin resistance and is associated with renal dysfunction. The present study sought to explore the relationship between TyG index and the incidence of contrast-induced nephropathy (CIN) in non-ST elevation acute coronary syndrome (NSTE-ACS) patients implanted with drug-eluting stents (DESs).

**Methods:**

A total of 1108 participants were recruited to the study and assigned to two groups based on occurrence of CIN. TyG index was calculated as ln [fasting triglycerides (mg/dL) × fasting blood glucose (mg/dL)/2]. Baseline characteristics and incidence of CIN were compared between the two groups. Logistic regression analysis was performed to evaluate the relationship between TyG index and CIN.

**Results:**

The results showed that 167 participants (15.1%) developed CIN. Subjects in the CIN group had a significantly higher TyG index compared with subjects in the non-CIN group (8.9 ± 0.7 vs. 9.3 ± 0.7, P<0.001). TyG index was significantly correlated with increased risk of CIN after adjusting for confounding factors irrespective of diabetes mellitus status and exhibited a J-shaped non-linear association. Subgroup analysis showed a significant gender difference in the relationship between TyG index and CIN. Receiver operating characteristic (ROC) curve analysis indicated that the risk assessment performance of TyG index was superior compared with other single metabolic indexes. Addition of TyG index to the baseline model increased the area under the curve from 0.713 (0.672-0.754) to 0.742 (0.702-0.782) and caused a reclassification improvement of 0.120 (0.092-0.149).

**Conclusion:**

The findings from the present study show that a high TyG index is significantly and independently associated with incidence of CIN in NSTE-ACS patients firstly implanted with DESs. Routine preoperative assessment of TyG index can alleviate CIN and TyG index provides a potential target for intervention in prevention of CIN.

## Introduction

Contrast-induced nephropathy (CIN) is defined as a decline in renal function occurring after intravascular administration of contrast agents (1). CIN is a common and potentially severe complication following coronary angiography and percutaneous coronary intervention (PCI). Therefore, CIN presents a major clinical challenge characterized by high mortality, irreversible kidney injury, and increased healthcare expenditure (2–4). Currently, no available adjuvant drugs are effective in treatment of CIN. Therefore, studies should explore methods for risk stratification and effective preventive strategies to improve prognosis of CIN patients.

Insulin resistance (IR) refers to decreased metabolic responsiveness and sensitivity of cells to insulin. IR presents as a shift of the insulin concentration-effect curve towards higher insulin concentrations. IR is highly correlated with occurrence of atherosclerosis, cardiovascular risk, and progression of chronic kidney disease (CKD) (5–9). Hyperinsulinemic-euglycemic clamp (HEC) test and homeostatic model assessment (HOMA) are effective methods for determination of IR (10, 11). However, clinical application of these methods is limited by high amount of time consumed, high cost, complexity, and high invasiveness. Recent studies reported a high correlation between HOMA-IR and HEC. Notably, triglyceride-glucose (TyG) index calculated based on fasting triglyceride (TG) and fasting blood glucose (FBG) is a powerful novel marker for IR in patients with and without diabetes mellitus (12, 13). In addition, TyG index is significantly associated with prevalence of diabetes, incidence of unstable carotid plaque, and adverse prognosis of cardiovascular diseases (CVD) (13–15).

To the best of our knowledge, the correlation between TyG index and incidence of CIN in non-ST elevation acute coronary syndrome (NSTE-ACS) patients who have undergone PCI has not been elucidated. Therefore, the present study was conducted to explore the underlying correlation between TyG index and CIN in NSTE-ACS patients who had undergone implantation with drug-eluting stents (DESs).

## Materials and Methods

### Study Population

**Figure 1:**
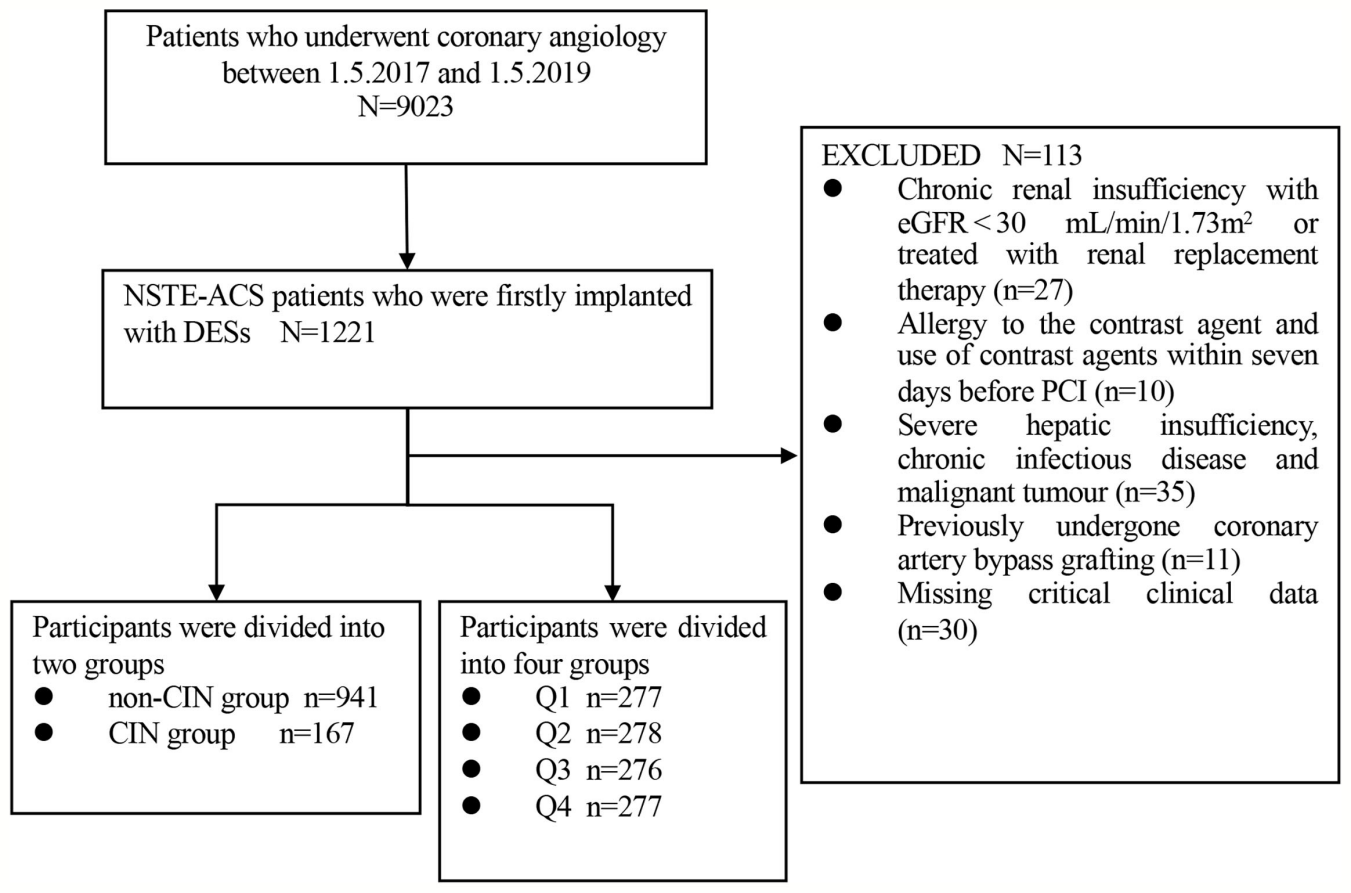
Flow diagram of participants selection.

This study was a single-center, observational, retrospective cohort study comprising NSTE-ACS patients who firstly underwent DESs implantation at the Department of Cardiology, Zhongda Hospital affiliated to Southeast University from May 2017 to May 2019. Exclusion criteria for the present study were as follows: (1) chronic renal insufficiency with estimated glomerular filtration rate (eGFR) < 30 mL/min/1.73 m^2^ or patients who had undergone renal replacement therapy; (2) allergy to the contrast agent; (3) use of contrast agents within seven days before PCI; (4) severe hepatic insufficiency, chronic infectious disease and malignant tumor; (5) previously undergone coronary artery bypass grafting; (6) missing key clinical data. A cohort of 1108 participants was included in the study for subsequent analyses (**Figure 1**).

### Groups

Participants were assigned two groups based on occurrence of CIN, namely: non-CIN group (n=941) and CIN group (n=167). Additionally, participants were further assigned to four quartiles as follows: Q1 (n=277, TyG index<8.44), Q2 (n=278, 8.44≤TyG index ≤ 8.85), Q3 (n=276, 8.86≤TyG index ≤ 9.29), and Q4 (n=277, TyG index≥9.30).

### Data Collection and Definitions

Patient demographic and clinical characteristics, including age, gender, smoking status, medical history, vital signs, laboratory indices, left ventricular ejection fraction (LVEF), angiographic variables, and medication used during hospitalization, were obtained from the medical record systems. Peripheral blood samples were obtained from the cubital vein after fasting overnight for at least 10 h. All laboratory indices were uniformly and preoperatively performed at the Department of Laboratory Medicine, Zhongda Hospital.

Body mass index (BMI) was calculated as the body mass divided by the square of patient height and presented in kg/m^2^. TyG index was determined using the following formula: ln [fasting TG (mg/dL)×FBG (mg/dL)/2]. Multiple vessel disease was defined as more than two main coronary branches with significant diameter stenosis (≥50%). Hypertension was defined as previous diagnosis or systolic blood pressure (SBP) ≥ 140mmHg or (and) diastolic blood pressure (DBP) ≥ 90mmHg presenting more than two times on different days. Criteria for diabetes mellitus were as follows: (1) self-reported diabetes mellitus previously diagnosed by a physician; (2) active treatment with hypoglycemic medication; (3) typical symptoms of diabetes with FBG ≥ 7.0mmol/L, and/or random blood glucose ≥ 11.1mmol/L, and/or 2-h blood glucose after oral glucose tolerance test  ≥ 11.1mmol/L, and/or hemoglobin A1c (HbA1c) ≥ 6.5%. CKD was defined as decreased kidney function characterized by eGFR less than 60 mL/min/1.73m^2^, or markers of kidney damage, or both, presenting for at least 3 months, regardless of the underlying cause (16). Diagnosis of NSTE-ACS was based on the criteria reported by the American College of Cardiology (17). CIN was defined as more than 25% or 44.2 umol/L (0.5 mg/dL) increase in serum creatinine (SCr) from baseline during the first 48 to 72 hours after contrast exposure (18). SCr levels were determined before and after PCI for 2-3 days. eGFR was calculated using the Chronic Kidney Disease Epidemiology Collaboration (CKD-EPI) equation (19). Mehran score was calculated by two investigators according to the scoring rules (20).

### Coronary Interventions and Medications

Two interventional cardiologists who were blind to the study protocol performed surgeries for all patients. DESs were implanted to the corresponding diseased vessels under the guide of coronary angiography results. All participants received a loading dose of aspirin (300mg) and clopidogrel (300mg) or ticagrelor (180mg) before the intervention. In addition, the medications such as beta-blockers, statins, angiotensin-converting enzyme inhibitors (ACEI) or angiotensin receptor blockers (ARB) were used.

### Statistical Analysis

Data analysis was performed using IBM SPSS Statistics 26.0 software (SPSS Inc. Chicago IL, USA), R version 3.6.1 (www.r-project.org), and Graphpad Prism (version 9.1.1 for macOS, GraphPad Software, LLC.). Continuous variables exhibiting normal distribution were expressed as mean ± standard deviation. Continuous variables that did not exhibit normal distribution were expressed as median (25th-75th interquartile range). Differences in characteristics between groups were compared using t-test, one-way analysis of variance, or rank-sum test. Categorical variables were reported as frequencies with percentages and compared by Chi-square test or Fisher’s exact test. Pearson’s or Spearman’s rank correlation coefficient analysis was conducted to explore correlations between TyG index and other baseline clinical data. Collinearity analysis was performed to explore variables that were significantly associated with the TyG index. Univariate and multivariate logistic regression analyses were conducted to evaluate whether TyG index was an independent risk factor for CIN. Variables with p<0.10 in univariate analysis were selected for use in multivariate analysis. FBG, TG, and significantly co-linear variables with TyG index were excluded. The results were expressed as odds ratios (ORs) with 95% confidence interval (CI). In addition, association between TyG index and CIN was verified using the generalized additive model with the smoothness of fit. Receiver operating characteristic (ROC) curve analysis was performed to evaluate diagnostic value of metabolic indexes. Area under the ROC curves (AUCs) was determined and compared by Z-test. Moreover, absolute net reclassification improvement (NRI) and integrated discrimination improvement (IDI) were used to evaluate advances in risk prediction quantification. Subgroup and interaction analyses for association between TyG index and CIN were performed to further assess the impact of TyG index on CIN. A two-sided P < 0.05 was considered statistically significant for all tests.

## Results

### Baseline Characteristics

A total of 1108 NSTE-ACS patients who had undergone DESs implantation were enrolled on the present study. The average age of the participants was 66.4 ± 10.4 years, average BMI was 24.9 ± 3.2 kg/m^2^, the female proportion was 37.5%, and the average TyG index was 8.9 ± 0.7 (**Table 1**). A total of 167 patients (15.1%) presented with CIN. Subjects in the CIN group had a significantly higher TyG index relative to subjects in the non-CIN group (9.3 ± 0.7 vs. 8.9 ± 0.7, P<0.001). Participants presenting with CIN exhibited a higher prevalence of hypertension, diabetes mellitus, non-ST elevation myocardial infarction (NSTEMI), higher levels of leukocyte count, SCr, preoperative eGFR, FBG, TG, total cholesterol (TC), and HbA1c, as well as higher proportion of multiple vessel disease and Gensini scores compared with non-CIN subjects. Moreover, participants in the CIN group received a higher dose of contrast agent but exhibited lower hydration rate and were more likely to undergo treatment with oral hypoglycemic drugs compared with subjects in non-CIN group. Participants were further assigned to four groups based on the quartile of TyG index. As shown in **Supplementary Table 1**, incidence of CIN increased gradually with increase in TyG index quartile (6.1%, 12.9%, 14.9%, and 20.0%, p=0.002). Furthermore, the results showed statistically significant differences in age, female proportion, BMI, SBP, DBP, hypertension, diabetes mellitus, NSTEMI, leukocyte count, hemoglobin, SCr, eGFR, FBG, TG, TC, low-density lipoprotein cholesterol (LDL-C) and HbA1c, three-vessel disease, multiple vessel disease, Gensini scores, oral hypoglycemic drugs, insulin, ACEI/ARB and beta-blockers among the four groups. Notably, no statistically significant differences were observed in the other baseline data among the four groups.

**Table 1:** Baseline characteristics of the total participants.

Variables	Total participants (N = 1108)	Non-CIN group (n = 941)	CIN group (n = 167)	P-value
TyG index	8.9 ± 0.7	8.9 ± 0.7	9.3 ± 0.7	<0.001
Age (years)	66.4 ± 10.4	66.5 ± 10.2	66.0 ± 11.4	0.666
Female, n (%)	416(37.5)	346 (36.8)	70 (41.9)	0.206
BMI (kg/m2)	24.9 ± 3.2	24.8 ± 3.2	25.8 ± 3.2	<0.001
SBP (mmHg)	136.7 ± 20.0	136.4 ± 20.0	138.1 ± 19.4	0.321
DBP (mmHg)	76.6 ± 12.8	76.6 ± 12.9	76.7 ± 11.8	0.975
Smoking, n (%)	298 (26.9)	251(26.7)	47 (28.1)	0.693
Hypertension, n (%)	801 (72.3)	668 (71.0)	133 (79.6)	0.021
Diabetes mellitus, n (%)	351 (31.7)	281 (29.9)	70 (41.9)	0.002
CKD, n (%)	39 (3.5)	30 (3.2)	9 (5.4)	0.155
Previous stroke, n (%)	251 (22.7)	209 (22.2)	42 (25.1)	0.403
NSTEMI, n (%)	235 (21.2)	189 (20.1)	46 (27.5)	0.026
Hydration, n (%)	851 (76.8)	748 (79.5)	103 (61.7)	<0.001
Diuretics, n (%)	179 (16.2)	128 (13.6)	51 (30.5)	<0.001
Dose of contrast agent, n (%)	100 (100,100)	100 (100,100)	100 (100,200)	<0.001
Biochemical indices				
Leukocyte count, ×10^9^/L	6.5 (5.5,7.9)	6.5 (5.4,7.8)	7.0 (5.7,8.3)	0.008
Hemoglobin, g/L	137.1 ± 16.6	137.4 ± 16.2	135.3 ± 18.7	0.170
SCr, umol/L	76 (66,89)	75(65,88)	80 (67,96)	0.005
eGFR, mL/min/1.73m^2^	84.9 (68.5,97.7)	85.8 (69.7,97.7)	80.1 (57.7,97.6)	0.007
Uric acid, μmol/L	353 (292,417)	350 (290,416)	365(299,422)	0.186
FBG, mmol/L	5.9 (5.2,7.5)	5.8 (5.2,7.2)	6.8(5.6,8.4)	<0.001
TG, mmol/L	1.4 (1.0,2.0)	1.4 (1.0,1.9)	1.8 (1.2,2.5)	0.020
TC, mmol/L	4.4 (3.7,5.1)	4.4 (3.7,5.1)	4.5 (3.8,5.6)	0.016
HDL-C, mmol/L	1.1 (1.0,1.3)	1.1 (0.9,1.3)	1.1 (0.9,1.3)	0.176
LDL-C, mmol/L	2.6 (2.0,3.3)	2.6 (2.0,3.3)	2.8 (2.1,3.4)	0.125
HbA1c (%)	6.1 (5.6,6.8)	6.0 (5.6,6.8)	6.4 (5.8,7.5)	<0.001
LVEF, (%)	67 (62,72)	67 (62,72)	68 (63,72)	0.523
Details of PCI				
Number of stents	1 (1,2)	1 (1,2)	1 (1,2)	0.107
Left main disease, n (%)	85 (7.7%)	78 (8.3)	7 (4.2)	0.067
Three-vessel disease, n (%)	451 (40.7)	375 (39.8)	76 (45.8)	0.149
Multiple vessel disease, n (%)	821 (74.1)	684 (72.7)	137 (82.0)	0.001
Gensini scores	54 (34,85)	52 (33,83)	59 (40,95)	0.012
Medication use				
Oral hypoglycemic drugs, n (%)	271 (24.5)	219 (23.3)	52 (31.1)	0.029
Insulin, n (%)	119 (10.7)	95 (10.1)	24 (14.4)	0.100
Aspirin, n (%)	1087 (98.1)	924 (98.2)	163 (97.6)	0.543
Clopidogrel/Ticagrelor, n (%)	1106 (99.8)	940 (99.9)	166 (99.4)	0.279
ACEI/ARB, n (%)	617 (55.7)	521 (55.4)	96 (57.5)	0.612
Beta-blockers, n (%)	864 (78.0)	728 (77.4)	136 (81.4)	0.242
Statin, n (%)	1105 (99.7)	939 (99.8)	166 (99.4)	0.388

CIN, contrast-induced nephropathy; TyG, triglyceride-glucose; BMI, body mass index; SBP, systolic blood pressure; DBP, diastolic blood pressure; CKD, chronic kidney disease; NSTEMI, non-ST elevation myocardial infarction; SCr, serum creatinine; eGFR, estimated glomerular filtration rate; FBG, fasting blood glucose; TG, triglyceride; TC, total cholesterol; HDL-C, high-density lipoprotein cholesterol; LDL-C, low-density lipoprotein cholesterol; HbA1c, hemoglobin A1c; LVEF, left ventricular ejection fraction; PCI, percutaneous coronary intervention; ACEI, angiotensin-converting enzyme inhibitors; ARB, angiotensin receptor blockers.

### Correlation Between TyG Index and Clinical Baseline Data

Pearson’s or Spearman’s rank correlation analysis was performed to explore the relationship between TyG index and clinical baseline data. As presented in **Table 2**, the TyG index was positively correlated with BMI as well as hemoglobin, TC, LDL-C, and HbA1c levels and negatively correlated with age and SCr (all p<0.05).

**Table 2:** Correlation between TyG index and potential risk factors.

Variables	Correlation coefficient (r)	P-value
Age	-0.205	<0.001
BMI	0.277	<0.001
Hemoglobin	0.130	<0.001
SCr	-0.079	0.009
Uric acid	0.058	0.055
TC	0.310	<0.001
HDL-C	-0.025	0.397
LDL-C	0.231	<0.001
HbA1c	0.425	<0.001
LVEF	0.015	0.615
Dose of contrast agent	0.038	0.211

BMI, body mass index; SCr, serum creatinine; TC, total cholesterol; HDL-C, high-density lipoprotein cholesterol; LDL-C, low-density lipoprotein cholesterol; HbA1c, hemoglobin A1c; LVEF, left ventricular ejection fraction.

### Independent Risk Factors for CIN After DESs Implantation

**Figure 2:**
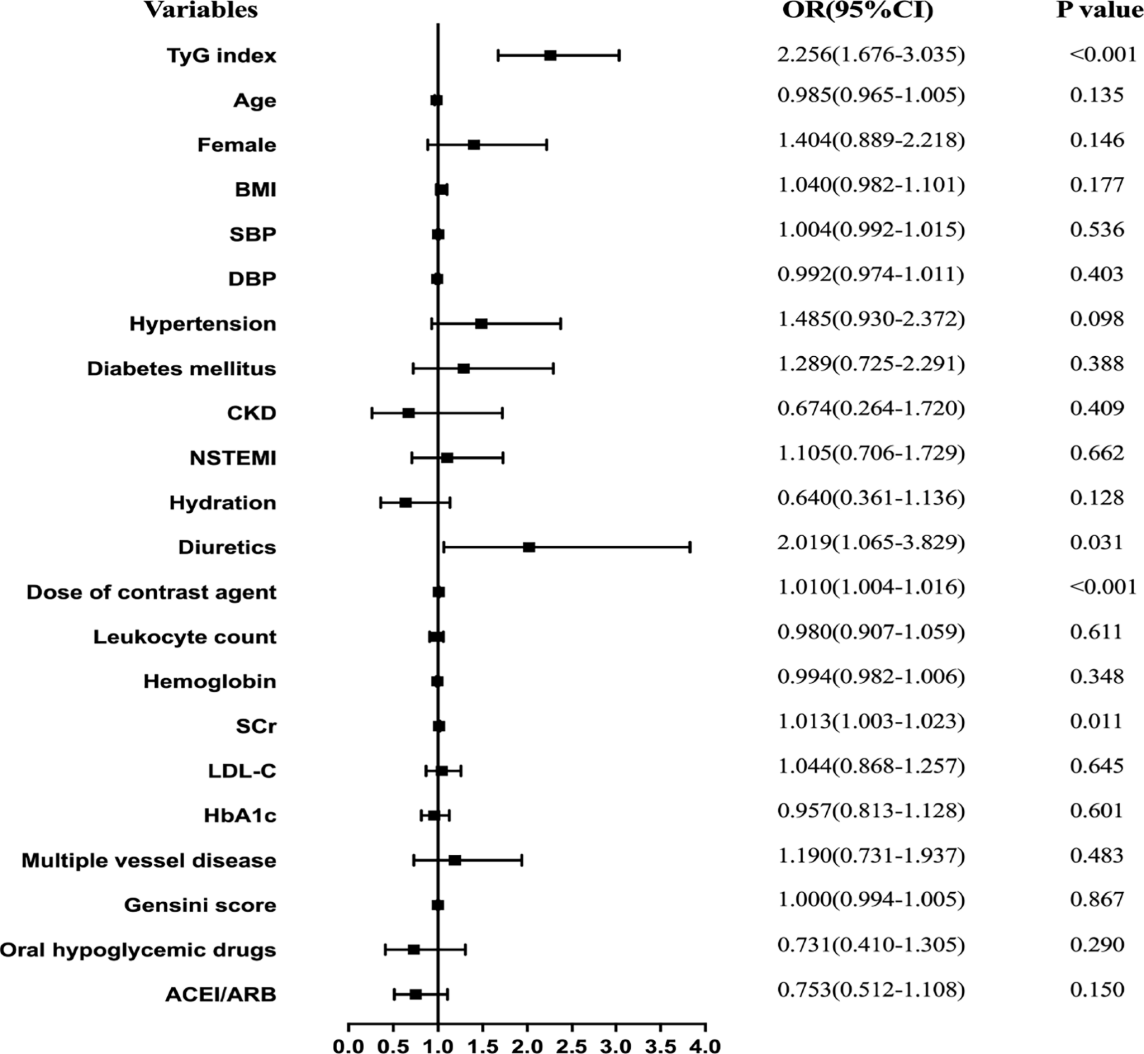
Forest plot of the multivariable logistic regression analysis exploring the association between the TyG index and CIN.

**Figure 3:**
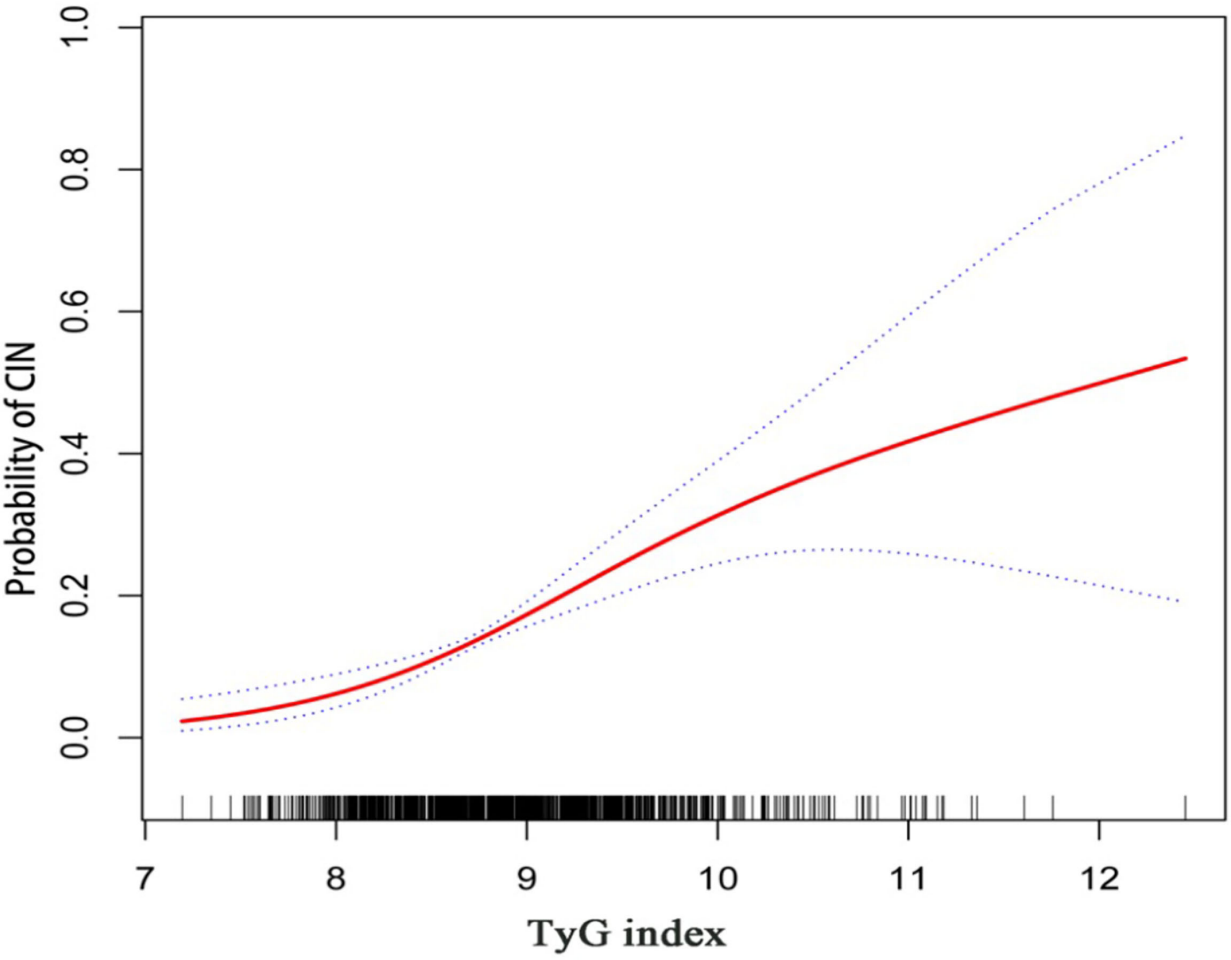
Generalized additive model plot for the relationship of the TyG index with CIN. Adjusted for confounding factors including age, female, BMI, SBP, DBP, hypertension, diabetes mellitus, CKD, NSTEMI, hydration, diuretics, the dose of contrast agent, leukocyte count, hemoglobin, SCr, LDL-C, HbA1c, multiple vessel disease, Gensini scores, oral hypoglycemic drugs, and ACEI/ARB.

Results for univariate and multivariate logistic regression analyses are presented in **Supplementary Table 2**. Univariate logistic regression analyses revealed that TyG index, BMI, hypertension, diabetes mellitus, NSTEMI, hydration, diuretics, the dose of contrast agent, leukocyte count, SCr, eGFR, FBG, TG, TC, HbA1c, multiple vessel disease, Gensini scores, and oral hypoglycemic drugs were risk factors for CIN. Collinearity analysis was then performed to explore whether these variables were closely related to TyG index. As shown in **Table 3**, TyG index had a high degree of collinearity with eGFR (Tolerance:0.087, VIF:11.429) and TC (Tolerance:0.094, VIF:10.667); thus were excluded from multivariate logistic regression analysis. Multivariate logistic regression analysis showed that TyG index (adjusted OR 2.256, 95%CI 1.676-3.035), SCr, diuretics, and dose of contrast agent were independent risk factors for development of CIN after DESs implantation, after adjusting for potential confounding risk factors (**Figure 2**). Moreover, the generalized additive model analysis showed a J-shaped dose-response relationship between TyG index and risk of CIN, after adjusting for confounding factors such as age, female, BMI, SBP, DBP, hypertension, diabetes mellitus, CKD, NSTEMI, hydration, diuretics, the dose of contrast agent, leukocyte count, hemoglobin, SCr, LDL-C, HbA1c, multiple vessel disease, Gensini scores, oral hypoglycemic drugs and ACEI/ARB (**Figure 3**). Further analysis of the threshold effect showed that the turning point of the curve was 9.701. The detailed outcome was presented in **Table 4**.

**Table 3:** Collinearity analysis of baseline data with TyG index.

	Unstandardized Coefficients		Standardized Coefficients	t	Sig.	Collinearity Statistics	
	B	Sth. Error	Beta			Tolerance	VIF
(Constant)	6.907	0.311		22.236	0.000		
Age	−0.002	0.001	−0.033	−1.721	0.086	0.375	2.664
Female	0.147	0.033	0.103	4.524	0.000	0.268	3.726
BMI	0.011	0.003	0.050	3.908	0.000	0.848	1.179
SBP	0.000	0.001	0.014	0.848	0.397	0.537	1.864
DBP	0.000	0.001	0.001	0.087	0.931	0.538	1.859
Hypertension	0.001	0.021	0.001	0.051	0.959	0.773	1.294
Diabetes mellitus	0.064	0.029	0.043	2.207	0.028	0.366	2.736
CKD	−0.017	0.052	−0.005	−0.328	0.743	0.739	1.352
NSTEMI	0.011	0.022	0.007	0.519	0.604	0.826	1.211
Hydration	0.045	0.028	0.027	1.616	0.106	0.484	2.066
Diuretics	−0.021	0.033	−0.011	−0.653	0.514	0.457	2.186
Dose of contrast agent	0.000	0.000	0.007	0.604	0.546	0.922	1.084
Leukocyte count	0.003	0.004	0.010	0.741	0.459	0.801	1.249
Hemoglobin	0.001	0.001	0.035	2.461	0.014	0.685	1.460
SCr	0.001	0.001	0.036	0.970	0.332	0.103	9.683
eGFR	0.001	0.001	0.026	0.657	0.512	0.087	11.429
FBG	0.104	0.004	0.404	25.114	0.000	0.539	1.855
TG	0.359	0.008	0.702	45.161	0.000	0.577	1.735
TC	−0.110	0.023	−0.188	−4.870	0.000	0.094	10.667
LDL-C	0.176	0.026	0.245	6.739	0.000	0.105	9.480
HbA1c	−0.013	0.009	−0.025	−1.419	0.156	0.443	2.256
Multiple vessel disease	0.030	0.021	0.019	1.415	0.157	0.775	1.290
Gensini scores	0.000	0.000	0.026	1.906	0.057	0.722	1.384
Oral hypoglycemic drugs	0.048	0.030	0.030	1.639	0.101	0.413	2.420
ACEI/ARB	−0.008	0.018	−0.006	−0.458	0.647	0.802	1.247

TyG, triglyceride-glucose; BMI, body mass index; SBP, systolic blood pressure; DBP, diastolic blood pressure; CKD, chronic kidney disease; NSTEMI, non-ST elevation myocardial infarction; SCr, serum creatinine; eGFR, estimated glomerular filtration rate; FBG, fasting blood glucose; TG, triglyceride; TC, total cholesterol; LDL-C, low-density lipoprotein cholesterol; HbA1c, hemoglobin A1c; ACEI, angiotensin-converting enzyme inhibitors; ARB, angiotensin receptor blockers.

**Table 4:** Threshold effect analysis of TyG index and CIN.

Outcome:	OR, 95%CI, P-value
Fitting model by a standard linear regression	2.256(1.676–3.035), p<0.001
Fitting model by two-piecewise linear regression	
Turning point (K)	9.701
<K segment effect 1	3.164(2.066–4.844), p<0.001
>K segment effect 2	1.177(0.610–2.268), p=0.627
The difference between the effect of 2 and 1	0.372(0.155–0.891), p=0.027
Log-likelihood ratio test	P=0.024

**Figure 4:**
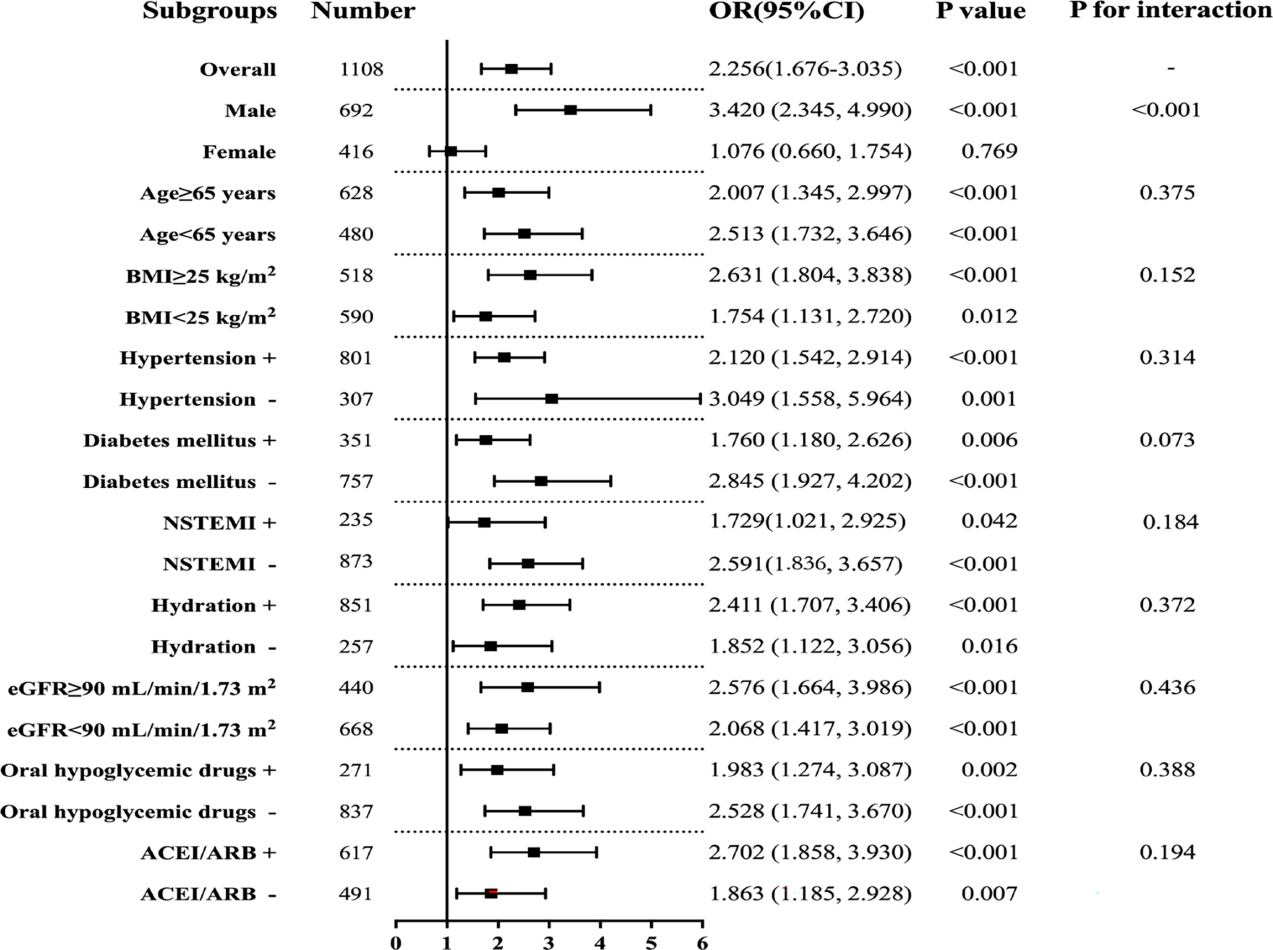
Subgroup analyses for the association between the TyG index and CIN. Adjusted for age, female, BMI, SBP, DBP, hypertension, diabetes mellitus, CKD, NSTEMI, hydration, diuretics, the dose of contrast agent, leukocyte count, haemoglobin, SCr, LDL-C, HbA1c, multiple vessel disease, Gensini scores, oral hypoglycemic drugs, and ACEI/ARB.

Subgroup analyses were conducted to further explore the relationship between TyG index and CIN (**Figure 4**). Results for subgroup analysis according to age, BMI, hypertension, diabetes mellitus, NSTEMI, hydration, eGFR, oral hypoglycemic drugs, or ACEI/ARB, were consistent with the overall group results. Notably, TyG index was an independent predictive factor for CIN in the male subgroup but not in the female subgroup. Elevated TyG index was associated with CIN regardless of diabetes mellitus status.

### Diagnostic Performance of TyG Index for CIN

**Figure 5:**
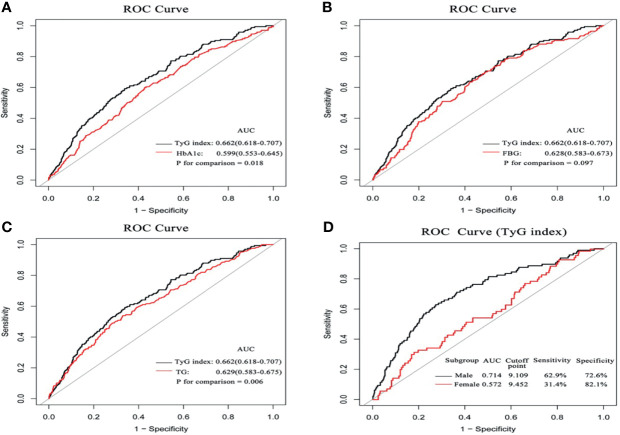
The comparison of diagnostic performance on the incidence of CIN between HbA1c **(A)** FBG **(B)** TG, **(C)**, and the impact of the TyG index between the male and female subgroups **(D)**.

ROC curve analysis showed that TyG index (AUC 0.662, 95%CI 0.618-0.707) exhibited the highest predictive value for CIN compared with HbA1c (AUC 0.599, 95%CI 0.553-0.645, P for comparison 0.018), FBG (AUC 0.628, 95%CI 0.583-0.673, P for comparison 0.097), and TG (AUC 0.629, 95%CI 0.583-0.675, P for comparison 0.006). The optimal cutoff point of TyG index was 9.043, with a sensitivity of 59.3% and specificity of 66.1%. Notably, AUC of TyG index for predicting CIN in the male subgroup was significantly higher relative to that in the female subgroup (0.714 vs. 0.572). Details of ROC curve analysis were exhibited in **Figure 5**.

### Incremental Effect of TyG Index in Predicting CIN

Further ROC curve analysis revealed that the AUC for predicting CIN was 0.713 in the baseline model, comprising age, female, BMI, SBP, DBP, hypertension, diabetes mellitus, CKD, NSTEMI, hydration, diuretics, the dose of contrast agent, leukocyte count, hemoglobin, SCr, LDL-C, multiple vessel disease, Gensini scores, oral hypoglycemic drugs and ACEI/ARB. With a NRI of 0.120 (P<0.001) and an IDI of 0.054 (P=0.122), the addition of TyG index to the baseline model significantly improved risk reclassification and discrimination for predicting CIN compared with addition of HbA1c, FBG and TG. Furthermore, adding the variable TyG to the Mehran model for predicting CIN further increased the predictive value of the model from 62.3% to 71.2%, whereas FBG increased the predictive value of the model from 62.3% to 65.0% (**Table 5**).

**Table 5:** Incremental effect of TyG index in predicting CIN.

Models	ROC analysis		NRI		IDI	
	AUC (95% CI)	P-value	Estimate (95% CI)	P-value	Estimate (95% CI)	P-value
Baseline model^a^	0.713 (0.672–0.754)		Ref.		Ref.	
+TyG index	0.742 (0.702–0.782)	0.027	0.120 (0.092–0.149)	<0.001	0.054 (−0.015 to 0.123)	0.122
+HbA1c	0.713 (0.672–0.754)	0.903	0.048 (0.016–0.080)	0.004	0.005 (−0.030 to 0.041)	0.764
+FBG	0.717 (0.676–0.758)	0.461	0.187 (0.162–0.212)	<0.001	0.008 (−0.056 to 0.071)	0.206
+TG	0.735 (0.695–0.774)	0.012	0.175 (0.150–0.201)	<0.001	0.020 (−0.048 to 0.087)	0.567
Mehran model	0.623 (0.579–0.668)		Ref.		Ref.	
+TyG index	0.712 (0.672–0.753)	<0.001	0.165 (0.132–0.198)	<0.001	0.153 (0.082–0.223)	<0.001
+FBG	0.650 (0.605–0.695)	0.015	0.229 (0.200–0.257)	<0.001	0.055 (−0.012 to 0.121)	0.105

a The baseline model includes age, female, BMI, SBP, DBP, hypertension, diabetes mellitus, CKD, NSTEMI, hydration, diuretics, dose of contrast agent, leukocyte count, hemoglobin, SCr, LDL-C, multiple vessel disease, Gensini scores, oral hypoglycemic drugs and ACEI/ARB. TyG, triglyceride-glucose; HbA1c, hemoglobin A1c; FBG, fasting blood glucose; TG, triglyceride; NRI, net reclassification improvement; IDI, integrated discrimination improvement.

## Discussion

The main findings of this study were as follows: (1) TyG index was significantly higher in patients with CIN compared with that in subjects without CIN; (2) TyG index was an independent risk factor for CIN after adjusting for confounding factors. Subgroup analysis indicated that TyG index exhibited a significant impact on CIN in the male subgroup compared with the female subgroup; (3) predictive value of TyG index for CIN was higher compared with the clinical value of other metabolic indicators, including HbA1c, FBG, and TG in predicting CIN risk; (4) addition of TyG index to the established models showed the most significant incremental effect on risk stratification for identifying CIN compared with other indicators. In summary, the results for the present study implied that patients with a higher TyG index, especially male patients, should be regarded as a high-risk CIN group, and effective preventive measures should be explored to reduce incidence of CIN in this group. To the best of our knowledge, this is the first study to evaluate the correlation between TyG index and CIN in NSTE-ACS patients who have undergone DESs implants.

IR is a complex and systemic glycolipid metabolic disorder characterized by hyperinsulinemia, hyperglycemia, and hyperlipidemia (21). IR plays an important role in development of diabetes, cardiovascular diseases, and renal insufficiency (22, 23). TyG index is derived from TG and FBG and is a reliable surrogate IR marker. Previous studies explored the relationship between TyG index and cardiovascular disease in multiple dimensions, such as the effect on pathology and clinical outcome, as well as in specific populations and the general population. Clinical studies report that elevated TyG index is significantly associated with subclinical atherosclerosis, unstable carotid plaque, and coronary artery calcium (15, 24, 25). Qian et al. conducted a prospective cohort study with a large sample size and the findings showed that TyG index was associated with an increased risk of CVD in the general Chinese population (26). Furthermore, elevated TyG index was independently associated with increased risk of cardiovascular events regardless of diabetes mellitus status. Notably, TyG index has higher clinical value in predicting cardiovascular risk in patients with ACS who underwent PCI compared with the clinical value of FPG and HbA1c (27).

CIN is the third cause of hospital-acquired acute kidney injury induced by renal perfusion insufficiency and nephrotoxic drugs. In addition, CIN affects patients undergoing cardiac interventional procedures (28). Therefore, it is imperative to explore the relationship between TyG index and CIN. Previous studies report that TyG index has a significant association with microalbuminuria, reduced eGFR, and diabetic kidney disease (29–31). However, studies have not explored the relationship between TyG index and CIN, especially in patients with ACS who have undergone PCI. The present study comprised NSTE-ACS patients with DESs implantation. The findings showed that TyG index is an independent risk factor for CIN, consistent with the observation of Qin et al. in patients with type 2 diabetes mellitus (32). In addition, TyG index exhibited the highest diagnostic value and risk stratification increment for CIN. A recent study conducted by Nusca et al. demonstrated that the GlyMehr score, a revised Mehran score obtained by including FBG in the original Mehran score showed a better predictive ability when compared with the Mehran score (33). In the present study, we found that TyG index provided a higher added value than FBG to the Mehran risk prediction model, and adding TyG rather than FBG to the Mehran score had higher predictive efficacy. Different enrolled populations and CIN definitions between the two studies may explain these findings. Moreover, further subgroup analysis demonstrated that the result was not affected by diabetes mellitus status, hypertension, BMI, or hypoglycemic treatment status. Notably, a high TyG index was significantly associated with increased risk of CIN in males but not in females. This observation can be attributed to significantly higher migration rate of adipose from subcutaneous to visceral depots in men compared with the rate in women, increasing IR development. Increased visceral obesity is implicated in the onset and progression of kidney dysfunction through hyperinsulinemia, inappropriate activation of the renin-angiotensin system, and oxidative stress in the kidney (34). A study by Ana et al. reported that gender plays an important role in the relationship between epigenetic age acceleration and obesity biomarkers. Male subjects exhibited a stronger association of epigenetic age acceleration with visceral adipose tissue mass and TyG index (35). This finding emphasizes the need for a gender-related risk management strategy to prevent CIN.

Currently validated preventative strategies for CIN include personalized hydration, reducing amount of contrast agent, and use of zero-contrast or ultra-low-contrast procedures (36). Some trials and meta-analyses of these trials report that these specific drugs significantly reduce the incidence of CIN. The Protective Effect of Rosuvastatin and Antiplatelet Therapy On contrast-induced acute kidney injury and myocardial damage in patients with Acute Coronary Syndrome (PRATO-ACS) trial explored the role of rosuvastatin in preventing CIN and the results showed that rosuvastatin (40 mg administered on admission, then 20 mg/day) reduced the incidence of CIN and improved short-term clinical outcomes (37). Statins are recommended as Class IIa preventative interventions in the European Society of Cardiology guidelines (38). In the current study, patients with NSTE-ACS were routinely treated with statins except for cases with contraindications. The findings showed no statistically significant difference in statin usage between the CIN group and non-CIN group. Furthermore, adequate management of high-risk factors such as hypertension, diabetes, and CKD is essential to reduce the risk of CIN. Findings from the Nephropathy in Diabetes type 2 (NID-2) study conducted in 14 Italian diabetes clinics showed that multi-factor intensive treatment significantly reduced the incidence of major adverse cardiovascular events in diabetic kidney disease patients with high cardiovascular risk compared with standard-of-care (39). This indicates that intensive treatment strategies based on major risk factors have high potential for CIN treatment in the future.

The correlation between TyG index and incidence of CIN can be attributed to pathogenetic association between IR and CIN. IR causes decreased podocyte number and podocyte foot process effacement, which are characteristics of nephropathy (34). The pathway to produce nitric oxide is impaired under IR state, whereas the pathway promoting vasoconstriction may not be affected or is activated, resulting in change in renal hemodynamics manifested as glomerular hyperfiltration and glomerular hypertension (40, 41).

The present study had several limitations. First, the current study was a single-center retrospective study. Thus it was difficult to fully elucidate the causal association between TyG index and CIN incidence. Secondly, FPG and TG levels were only determined at admission, and there was no further analysis conducted during the follow-up period. Third, some potential confounding factors such as the types of hypoglycemic drugs and duration of diabetes were not adjusted for in the multiple regression analysis. Fourth, the level of evidence supporting the value TyG index in predicting occurrence of CIN was reduced owing to the lack of comparison with HOMA-IR or HEC. Furthermore, this study used only one of the current definitions of CIN; thus, the results might potentially not be repeatable using other CIN diagnostic criteria. Finally, the study comprised intermediate-low risk NSTE-ACS patients who had firstly undergone DESs implantation in China. For high-risk NSTEMI or STEMI patients requiring primary PCI, the results of this study may not be applicable; thus, further studies should explore the application of these results in other populations.

## Conclusion

The results of the present study showed that high TyG index is independently associated with an increased risk of CIN in NSTE-ACS patients implanted with DES. Moreover, the addition of TyG index to the established models increased the predictive value for CIN. This indicates that elevated TyG index is a potential marker for high-risk CIN group.

## Data Availability Statement

The datasets used in the study are available from the corresponding author upon reasonable request.

## Ethics Statement

The studies involving human participants were reviewed and approved by the Clinical Research Ethics Committee of the Affiliated Zhongda Hospital of Southeast University. The patients/participants provided their written informed consent to participate in this study.

## Author Contributions

ML, GY, and CT conceived and designed the experiments and wrote the manuscript. ML, LL, and YuQ performed the experiments and analyzed the data. EL, DW, and YoQ took the quality control of data and critically revised the manuscript. All authors read and approved the final manuscript.

## Funding

This study was supported by the National Natural Science Foundation of China (81970237 and 82170433).

## Conflict of Interest

The authors declare that the research was conducted in the absence of any commercial or financial relationships that could be construed as a potential conflict of interest.

## Publisher’s Note

All claims expressed in this article are solely those of the authors and do not necessarily represent those of their affiliated organizations, or those of the publisher, the editors and the reviewers. Any product that may be evaluated in this article, or claim that may be made by its manufacturer, is not guaranteed or endorsed by the publisher.

## Abbreviations

CIN, contrast-induced nephropathy; TyG, triglyceride-glucose; NSTE-ACS, non-ST elevation acute coronary syndrome; PCI, percutaneous coronary intervention; DESs, drug-eluting stents; ROC, receiver operating characteristic; IR, insulin resistance; HEC, hyperinsulinemic-euglycemic clamp; CVD, cardiovascular diseases; BMI, body mass index; SBP, systolic blood pressure; DBP, diastolic blood pressure; CKD, chronic kidney disease; ORs, odds ratios; CI, confidence interval; NRI, net reclassification improvement; IDI, integrated discrimination improvement; NSTEMI, non-ST elevation myocardial infarction; SCr, serum creatinine; eGFR, estimated glomerular filtration rate; FBG, fasting blood glucose; TG, triglyceride; TC, total cholesterol; HDL-C, high-density lipoprotein cholesterol; LDL-C, low-density lipoprotein cholesterol; HbA1c, hemoglobin A1c; LVEF, left ventricular ejection fraction; PCI percutaneous coronary intervention; ACEI, angiotensin-converting enzyme inhibitors; ARB, angiotensin receptor blockers.
