# Supplementary material for: Elevated TyG Index Predicts Incidence of Contrast-Induced Nephropathy: A Retrospective Cohort Study in NSTE-ACS Patients Implanted With DESs
Source: Front Endocrinol (Lausanne). 2022 Feb 22;13:817176. doi: 10.3389/fendo.2022.817176 (PMC8901499; doi:10.3389/fendo.2022.817176)
Supplement: Supplementary file 1 [file DataSheet_1.docx]

**Supplementary Table 1. Baseline characteristics of all participants according to the TyG index quartile**

| Variable | Q1(n=277) | Q2(n=278) | Q3(n=276) | Q4(n=277) | P-value |
| --- | --- | --- | --- | --- | --- |
| TyG index | 8.1±0.2 | 8.7±0.1 | 9.1±0.1 | 9.8±0.5 | <0.001 |
| CIN | 17(6.1) | 36(12.9) | 41(14.9) | 72(26.0) | <0.001 |
| Age (years) | 68.9±10.2 | 67.4±10.0 | 65.4±10.3 | 63.9±10.5 | <0.001 |
| Female, n (%) | 74(26.7) | 107(38.5) | 123(44.6) | 112(40.4) | <0.001 |
| BMI (kg/m2) | 23.6±3.2 | 25.0±3.2 | 25.1±2.9 | 26.0±3.2 | <0.001 |
| SBP (mmHg) | 134.9±21.7 | 135.1±17.5 | 138.8±20.0 | 137.9±20.1 | 0.046 |
| DBP (mmHg) | 74.5±13.6 | 77.1±12.5 | 77.8±12.5 | 77.2±12.3 | 0.013 |
| Smoking, n (%) | 85(30.7) | 73(26.3) | 60(21.7) | 80(28.9) | 0.095 |
| Hypertension, n (%) | 184(66.4) | 193(69.4) | 214(77.5) | 210(75.8) | 0.010 |
| Diabetes mellitus, n (%) | 43(15.5) | 54(19.4) | 102(37.0) | 152(54.9) | <0.001 |
| CKD, n (%) | 8(2.9) | 15(5.4) | 6(2.2) | 10(3.6) | 0.196 |
| Previous stroke, n (%) | 65(23.5) | 73(26.3) | 66(23.9) | 47(17.0) | 0.057 |
| NSTEMI, n (%) | 47(17.0) | 54(19.4) | 61(22.1) | 73(26.4) | 0.045 |
| Hydration, n (%) | 203(73.3) | 209(75.2) | 221(80.1) | 218(78.7) | 0.208 |
| Diuretics, n (%) | 48(17.3) | 50(18.0) | 39(14.1) | 42(15.2) | 0.571 |
| Dose of contrast agent | 100(100,100) | 100(100,100) | 100(100,100) | 100(100,100) | 0.493 |
| Biochemical indices |  |  |  |  |  |
| Leukocyte count, 10^9^/L | 6.1(5.2,7.4) | 6.5(5.4,7.8) | 6.6(5.5,7.9) | 7.0(5.9,8.5) | <0.001 |
| Hemoglobin, g/L | 134.8±16.4 | 135.9±16.5 | 137.4±16.3 | 140.2±16.7 | 0.001 |
| SCr, umol/L | 78(66,93) | 79(67,92) | 74(62,87) | 74(66,86) | 0.002 |
| eGFR, mL/min/1.73m^2^ | 83.4(66.0,96.9) | 79.1(66.7,94.0) | 86.1(71.7,98.9) | 88.3(70.8,103.0) | 0.003 |
| Uric acid, μmol/L | 352(281,414) | 348(294,422) | 352(291,414) | 364(298,421) | 0.419 |
| Glucose, mmol/L | 5.2(4.8,5.9) | 5.6(5.1,6.5) | 6.0(5.4,7.5) | 8.1(6.6,10.8) | <0.001 |
| TG, mmol/L | 0.8(0.7,1.0) | 1.3(1.1,1.4) | 1.7(1.4,2.0) | 2.6(2.1,3.3) | <0.001 |
| TC, mmol/L | 4.0(3.3,4.7) | 4.2(3.5,4.9) | 4.6(3.9,5.3) | 4.8(4.2,5.8) | <0.001 |
| LDL-C, mmol/L | 2.3(1.8,2.9) | 2.6(1.9,3.3) | 2.9(2.2,3.4) | 2.9(2.3,3.6) | <0.001 |
| HbA1c (%) | 5.7(5.4,6.2) | 5.9(5.5,6.3) | 6.1(5.7,6.9) | 6.9(6.1,8.2) | <0.001 |
| LVEF, (%) | 66(61,72) | 67(62,71) | 68(63,72) | 67(61,72) | 0.222 |
| Coronary angiography |  |  |  |  |  |
| Number of stents | 1(1,2) | 1(1,2) | 1(1,2) | 1(1,2) | 0.623 |
| Left main disease, n (%) | 19(6.9) | 20(7.2) | 22(8.0) | 24(8.7) | 0.857 |
| Three vessel disease, n (%) | 95(34.3) | 110(39.6) | 106(38.4) | 140(50.5) | 0.001 |
| Multiple vessel disease, n (%) | 189(68.2) | 207(74.5) | 204(73.9) | 221(79.8) | 0.022 |
| Gensini scores | 49(31,80) | 54(33,80) | 53(35,90) | 59(36,90) | 0.011 |
| Medications use |  |  |  |  |  |
| Oral hypoglycemic drugs, n(%) | 30(10.8) | 33(11.9) | 81(29.3) | 127(45.8) | <0.001 |
| Insulin, n (%) | 13(4.7) | 13(4.7) | 30(10.9) | 63(22.7) | <0.001 |
| Aspirin, n (%) | 271(97.8) | 271(97.5) | 274(99.3) | 271(97.8) | 0.418 |
| Clopidogrel/ Ticagrelor, n (%) | 276(99.6) | 277(99.6) | 276(100) | 277(100) | 0.573 |
| ACEI/ARB, n (%) | 130(46.9) | 161(57.9) | 163(59.1) | 163(58.8) | 0.009 |
| Beta-blockers, n (%) | 195(70.4) | 224(80.6) | 223(80.8) | 222(80.1) | 0.006 |
| Statin, n (%) | 276(99.6) | 276(99.3) | 276(100.0) | 277(100.0) | 0.301 |

TyG triglyceride-glucose, BMI body mass index, SBP systolic blood pressure, DBP diastolic blood pressure, CKD chronic kidney disease, NSTEMI non-ST elevation myocardial infarction, SCr serum creatinine, eGFR estimated glomerular filtration rate, FBG fasting blood glucose, TG triglyceride, TC total cholesterol, HDL-C high-density lipoprotein cholesterol, LDL-C low-density lipoprotein cholesterol, HbA1c hemoglobin A1c, LVEF left ventricular ejection fraction, PCI percutaneous coronary intervention, ACEI angiotensin-converting enzyme inhibitors, ARB angiotensin receptor blockers.

**Supplementary Table 2. Univariate and multivariate logistic analysis for predicting CIN after DESs implantation**

|  | Univariate analysis | | |  | Multivariate analysis | | |
| --- | --- | --- | --- | --- | --- | --- | --- |
|  | OR | 95%CI | P-value |  | OR | 95%CI | P-value |
| TyG index | 2.129 | 1.695-2.673 | <0.001 |  | 2.256 | 1.676-3.035 | <0.001 |
| Age | 0.996 | 0.981-1.012 | 0.639 |  | 0.985 | 0.965-1.005 | 0.135 |
| Female | 1.241 | 0.888-1.734 | 0.206 |  | 1.404 | 0.889-2.218 | 0.146 |
| BMI | 1.102 | 1.047-1.159 | <0.001 |  | 1.040 | 0.982-1.101 | 0.177 |
| SBP | 1.004 | 0.996-1.012 | 0.321 |  | 1.004 | 0.992-1.015 | 0.536 |
| DBP | 1.000 | 0.987-1.013 | 0.975 |  | 0.992 | 0.974-1.011 | 0.403 |
| Smoking | 1.077 | 0.746-1.554 | 0.693 |  |  |  |  |
| Hypertension | 1.599 | 1.069-2.390 | 0.022 |  | 1.485 | 0.930-2.372 | 0.098 |
| Diabetes mellitus | 1.695 | 1.209-2.376 | 0.002 |  | 1.289 | 0.725-2.291 | 0.388 |
| CKD | 1.730 | 0.806-3.713 | 0.160 |  | 0.674 | 0.264-1.720 | 0.409 |
| Previous stroke | 1.177 | 0.803-1.724 | 0.403 |  |  |  |  |
| NSTEMI | 1.513 | 1.040-2.201 | 0.031 |  | 1.105 | 0.706-1.729 | 0.662 |
| Hydration | 0.415 | 0.293-0.589 | <0.001 |  | 0.640 | 0.361-1.136 | 0.128 |
| Diuretics | 2.792 | 1.913-4.077 | <0.001 |  | 2.019 | 1.065-3.829 | 0.031 |
| Dose of contrast agent | 1.009 | 1.004-1.014 | <0.001 |  | 1.010 | 1.004-1.016 | <0.001 |
| Biochemical indices |  |  |  |  |  |  |  |
| Leukocyte count | 1.078 | 1.011-1.150 | 0.022 |  | 0.980 | 0.907-1.059 | 0.611 |
| Hemoglobin | 0.992 | 0.983-1.002 | 0.129 |  | 0.994 | 0.982-1.006 | 0.348 |
| SCr | 1.010 | 1.003-1.017 | 0.006 |  | 1.013 | 1.003-1.023 | 0.011 |
| eGFR | 0.988 | 0.980-0.996 | 0.003 |  |  |  |  |
| Uric acid | 1.001 | 0.999-1.002 | 0.437 |  |  |  |  |
| FBG | 1.110 | 1.055-1.169 | <0.001 |  |  |  |  |
| TG | 1.280 | 1.153-1.421 | <0.001 |  |  |  |  |
| TC | 1.176 | 1.030-1.343 | 0.017 |  |  |  |  |
| HDL-C | 0.838 | 0.535-1.314 | 0.441 |  |  |  |  |
| LDL-C | 1.138 | 0.965-1.343 | 0.125 |  | 1.044 | 0.868-1.257 | 0.645 |
| HbA1c | 1.214 | 1.091-1.350 | <0.001 |  | 0.957 | 0.813-1.128 | 0.601 |
| LVEF | 0.998 | 0.984-1.013 | 0.818 |  |  |  |  |
| Details of PCI |  |  |  |  |  |  |  |
| Number of stents | 1.180 | 0.941-1.480 | 0.152 |  |  |  |  |
| Left main disease | 0.784 | 0.229-1.690 | 0.132 |  |  |  |  |
| Three-vessel disease | 1.261 | 0.905-1.756 | 0.171 |  |  |  |  |
| Multiple vessel disease | 1.716 | 1.127-2.612 | 0.012 |  | 1.190 | 0.731-1.937 | 0.483 |
| Gensini scores | 1.006 | 1.001-1.010 | 0.011 |  | 1.000 | 0.994-1.005 | 0.867 |
| Medications use |  |  |  |  |  |  |  |
| Oral hypoglycemic drugs | 1.491 | 1.039-2.138 | 0.030 |  | 0.731 | 0.410-1.305 | 0.290 |
| Insulin | 1.495 | 0.923-2.419 | 0.102 |  |  |  |  |
| Aspirin | 0.750 | 0.249-2.257 | 0.608 |  |  |  |  |
| Clopidogrel/ Ticagrelor | 0.177 | 0.011-2.837 | 0.221 |  |  |  |  |
| ACEI/ARB | 1.090 | 0.782-1.520 | 0.612 |  | 0.753 | 0.512-1.108 | 0.150 |
| Beta-blockers | 1.284 | 0.844-1.951 | 0.243 |  |  |  |  |
| Statin | 0.354 | 0.032-3.922 | 0.397 |  |  |  |  |

TyG triglyceride-glucose, BMI body mass index, SBP systolic blood pressure, DBP diastolic blood pressure, CKD chronic kidney disease, NSTEMI non-ST elevation myocardial infarction, SCr serum creatinine, eGFR estimated glomerular filtration rate, FBG fasting blood glucose, TG triglyceride, TC total cholesterol, HDL-C high-density lipoprotein cholesterol, LDL-C low-density lipoprotein cholesterol, HbA1c hemoglobin A1c, LVEF left ventricular ejection fraction, PCI percutaneous coronary intervention, ACEI angiotensin-converting enzyme inhibitors, ARB angiotensin receptor blockers.
